# Prognostic value of the fibrinogen albumin ratio index (FARI) in nasopharyngeal carcinoma patients undergoing radiotherapy

**DOI:** 10.1038/s41598-023-48029-0

**Published:** 2023-11-23

**Authors:** Chao Deng, Sujuan Zhang, Jie Ling, Zui Chen, Yuhua Feng, Yangchun Xie, Xianling Liu, Chunhong Hu, Tao Hou

**Affiliations:** Department of Oncology, The Second Xiangya Hospital, Central South University, Changsha, 410011 Hunan People’s Republic of China

**Keywords:** Cancer, Head and neck cancer, Cancer

## Abstract

There is mounting evidence that malnutrition and systemic inflammation status are involved in the prognosis of various cancers. In this study, we aimed to evaluate the prognostic value of the pretreatment fibrinogen-albumin ratio index (FARI) in nasopharyngeal carcinoma (NPC) patients receiving definite radiotherapy. NPC patients who received definite radiotherapy between January 2013 and December 2019 were included. A receiver operating characteristic (ROC) curve was used to determine the optimal cutoff value. The clinicopathological characteristics of the patients were compared via the Chi-square test. Survival curves were analyzed by the Kaplan‒Meier method. The prognostic factors were evaluated by univariate and multivariate analyses via Cox hazards regression analysis. A total of 225 patients were enrolled, and the median follow-up time was 48.5 months. High FARI was correlated with worse ECOG score (p = 0.003), higher EBV-DNA titer (p = 0.047), and more advanced clinical stage (p < 0.001). In the multivariable analysis, FARI independently predicted OS (HR 2.399, 95% CI 1.294–4.450, P < 0.001), PFS (HR 2.085, 95% CI 1.200–3.625, P = 0.009), and DMFS (HR 2.527, 95% CI 1.288–4.958, P < 0.001). The current findings suggest that a high pretreatment FARI is an independent predictor of OS, PFS and DMFS in NPC patients undergoing definite radiotherapy.

## Introduction

Nasopharyngeal carcinoma (NPC) is the most common head and neck malignancy in southern China and southeast Asia^1^. Radiotherapy is the cornerstone of treatment due to the special anatomical site and radiation sensitivity of the tumor. With the development of radiotherapy and medical treatments, the prognosis of NPC patients, especially locally advanced NPC patients, has significantly improved^2^. However, approximately 10% of patients will still suffer from relapse, and 20% will suffer from metastasis^3^. Thus, exploring effective and economical biomarkers to predict the prognosis of NPC patients and facilitate patient stratification and treatment modification is urgently needed.

Promotion of inflammation and deregulation of metabolism are widely accepted as a hallmark of cancer^4^. Increasing evidence has shown that the inflammatory and nutritional status of the host is closely related to treatment sensitivity and prognosis. Albumin is the classical biomarker for nutritional status, and previous studies have shown that it is a prognostic factor in various malignancies, including hepatocellular carcinoma, esophageal carcinoma and non-small cell lung cancer^5–7^. Fibrinogen is an important coagulation factor in the blood. However, increasing evidence has shown that fibrinogen also acts as an inflammatory mediator that is involved in systemic inflammation, cancer cell adhesion, and cancer progression^8,9^. Basic studies have shown that fibrinogen could affect innate immunity by inhibiting the function of macrophages and NK cells. The correlation between plasma fibrinogen level and prognosis has been reported in several kinds of cancers^10–12^. FARI is an index that reflects measures of both albumin and fibrinogen. It has been reported that FARI is an independent prognostic factor in several kinds of cancers, including head and neck squamous cell carcinoma^13^, gastric cancer^14^, cholangiocarcinoma^15^, and pancreatic ductal adenocarcinoma^16^. However, the prognostic role of FARI in NPC patients has not yet been reported.

In the present study, we retrospectively analyzed the prognostic value of FARI, as well as other nutritional and inflammation indices, in a cohort of NPC patients.

## Methods and materials

### Patients

The data from patients diagnosed with NPC who underwent definitive radiotherapy, with or without chemotherapy, at the Second Xiangya Hospital, Central South University from January 2013 to December 2019 were retrospectively analyzed. The exclusion criteria were as follows: (1) patients with incomplete clinical-pathological data; (2) patients with missing laboratory test results; (3) patients with incomplete follow-up data; (4) patients with a history of chronic inflammatory diseases such as inflammatory bowel disease; and (5) patients with recent acute infectious diseases. The research was carried out in line with the Declaration of Helsinki and approved by the Ethics Committee of the Second Xiangya Hospital of Central South University, and informed consent was waived.

### Data collection

The demographic and clinical pathological data and laboratory results were obtained from the hospital medical records system. Data regarding patients’ age, sex, Eastern Cooperative Oncology Group performance status (ECOG PS) scores, T stage, N stage, clinical stage, treatment modality, height, body weight, neutrophil, lymphocyte, platelet, monocyte, serum albumin, plasma fibrinogen, and EBV-DNA were collected. The plasma fibrinogen was tested via the Clauss method, and the serum albumin was tested via the bromocresol green method. The method of quality control is to use two levels of quality control products for machine testing, and the error should be within the range set by the laboratory. The FARI was calculated as follows: FARI = fibrinogen/albumin.

### RT procedure

All patients were treated with intensity-modulated radiation therapy (IMRT). The gross tumor volume and clinical tumor volume were defined according to the guidelines^17^. The prescribed dose was 70 Gy/2.12 Gy/33 F for PGTV, 60 Gy/1.82 Gy/33 F for PTV1, and 54 Gy/1.64 Gy/33 F for PTV2. Radiotherapy was delivered once daily 5 days per week. Concurrent chemotherapy (cisplatin or nedaplatin) and targeted therapy (nimotuzumab) were administered according to the stage and tolerability of the patients.

### Follow-up

Patients were followed up by telephone review or inpatient and outpatient medical records. The last follow-up date was April 30, 2023. Overall survival (OS) was calculated from the date of diagnosis to the date of death from any cause or to the last date of follow-up. Progression-free survival (PFS) was defined between the date of diagnosis and the date of disease progression or death.

### Statistical analysis

SPSS statistical software (version 22.0; SPSS Inc., Chicago, IL, USA) was used for data analysis. Receiver operating characteristic (ROC) curves were used to calculate the cutoff value for FARI. The Chi-square test was used to analyze the relationship between FARI and clinicopathological features. The Kaplan–Meier method was used to calculate survival curves. Multivariate analysis was based on the Cox regression model. A two-sided p value < 0.05 was considered statistically significant.

### Ethics statement

This research was carried out in line with the Declaration of Helsinki and approved by the Ethics Committee of the Second Xiangya Hospital of Central South University. The Ethics Committee of the Second Xiangya Hospital of Central South University waived the requirement of written informed consent.

## Results

### Patient characteristics

The characteristics of 225 enrolled NPC patients are presented in Table 1. The median age was 49 years (range: 15–71 years). One hundred and sixty-two out of 225 (72.0%) were male. One hundred thirteen patients (59.1%) had an ECOG score of 0. The mean body mass index (BMI) was 23.56 ± 3.38 kg/m^2^, with 8.4% of patients being underweight. Sixty-one patients (27.1%) had a positive EBV-DNA test, which was defined as more than 400 copies/ml, and 81 patients (36.0%) had a negative EBV-DNA test. However, there were 83 patients (36.9%) whose EBV-DNA status was unknown. Thirty-four patients (15.1%) had stage II tumors, 132 patients (58.7%) had stage III tumors, and 59 patients (26.2%) had stage IVa tumors according to the AJCC 8th edition staging system. Among all the patients, 71 (31.6%) received concurrent chemoradiotherapy (CCRT), and the rest of the patients received radiotherapy or radiotherapy concurrent with other drugs (nimotuzumab, etc.). With a median follow-up time of 48.5 months (range: 5.7–117.3 months), 19 (8.4%) patients suffered from recurrence, 46 (20.4%) patients had experienced metastasis, and 53 (23.6%) patients died.

**Table 1:** The baseline clinicopathological characteristics of NPC patients (n = 225).

Characteristics	Number (%)
Age	
Median	49
Range	15–71
Sex	
Male	162 (72.0%)
Female	63 (28.0%)
ECOG PS	
0	133 (59.1%)
1	89 (39.6%)
2	3 (1.3%)
T stage	
1	31 (13.8%)
2	86 (38.2%)
3	72 (32.0%)
4	36 (16.0%)
N stage	
0	9 (4.0%)
1	48 (21.3%)
2	143 (63.6%)
3	25 (11.1%)
Clinical stage	
II	34 (15.1%)
III	132 (58.7%)
IVa	59 (26.2%)
BMI (kg/m^2^)	
< 18.5	19 (8.4%)
18.5–24.0	99 (44.0%)
> 24.0	107 (47.6%)
Mean ± SD	23.56 ± 3.38
EBV-DNA (copy/ml)	
< 400	81 (36.0%)
≥ 400	61 (27.1%)
Unknown	83 (36.9%)
Treatment	
CCRT	71 (31.6%)
Non-CCRT	154 (68.4%)
Relapse	
Yes	19 (8.4%)
No	206 (91.6%)
Metastasis	
Yes	46 (20.4%)
No	179 (79.6%)
Death	
Yes	53 (23.6%)
No	172 (76.4%)

*ECOG PS* eastern clinical oncology group performance status, *BMI* body mass index, *CCRT* concurrent chemoradiation therapy.

### Cutoff value of FARI and the association with clinicopathological characteristics

**Figure 1:**
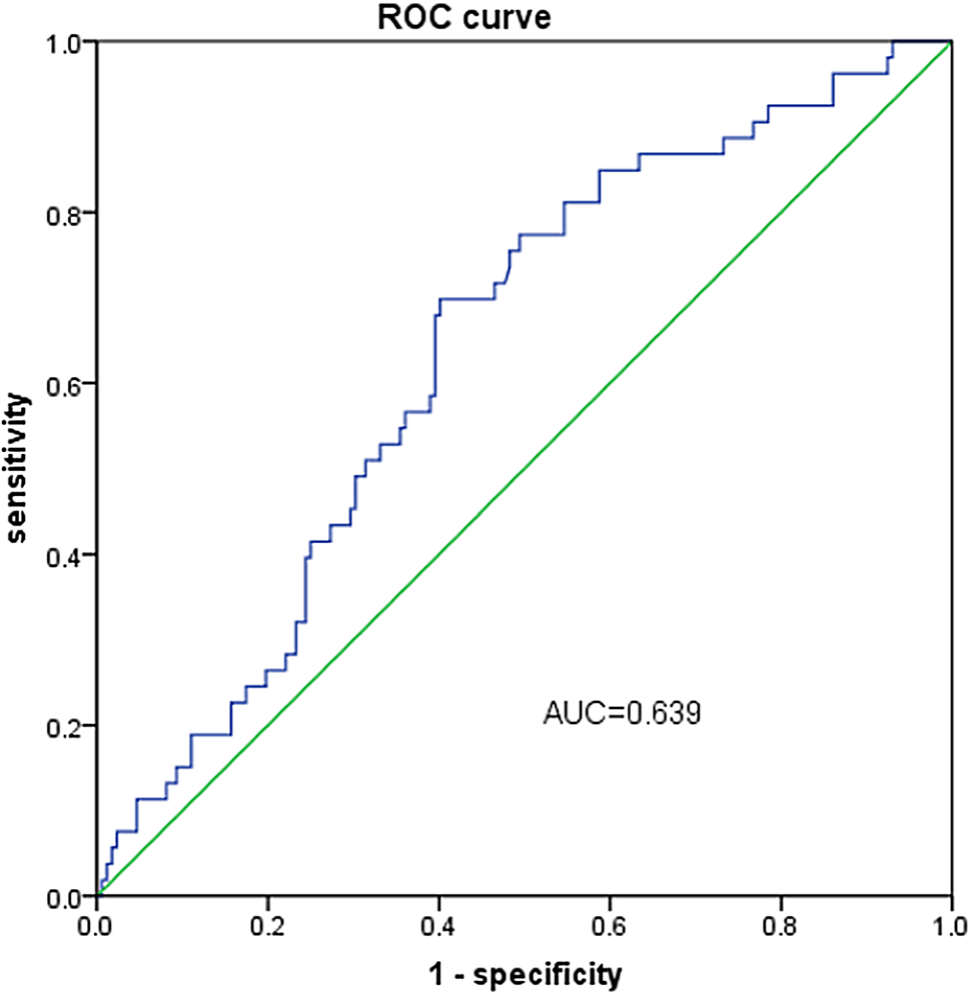
ROC curve of FARI in NPC patients.

By setting overall survival status as the endpoint, ROC analysis was used to determine the cutoff value of FARI, and the optimal cutoff value of FARI was 0.0761 (Fig. 1). All patients were categorized into a high FARI group and a low FARI group according to the cutoff value. The association between FARI and the clinicopathological characteristics of the patients is shown in Table 2. High FARI was associated with worse ECOG score (p = 0.003), higher EBV-DNA titer (p = 0.047), and more advanced T stage and clinical stage (p < 0.001). FARI status was not associated with age, sex, BMI, N stage or treatment modality (all p > 0.05).

**Table 2:** Clinicopathological characteristics of 225 patients with NPC.

Variables	Low FARI (%)	High FARI (%)	P value
Age			
≤ 60	107 (89.9%)	89 (84%)	0.129
> 60	12 (10.1%)	17 (16%)	
Sex			
Male	92 (77.3%)	70 (66%)	0.074
Female	27 (22.7%)	36 (34%)	
ECOG PS			
0	83 (69.7%)	50 (47.2%)	**0.003**
1	35 (29.4%)	54 (50.9%)	
2	1 (0.8%)	2 (1.9%)	
BMI			
< 18.5	11 (9.2%)	8 (7.5%)	0.497
18.5–24	48 (40.3%)	51 (48.1%)	
≥ 24	60 (50.4%)	47 (44.3%)	
EBV-DNA (n = 142)			
< 400	51 (64.6%)	30 (47.6%)	**0.043**
≥ 400	28 (35.4%)	33 (52.4%)	
T stage			
1–2	77 (64.7%)	40 (37.7%)	** < 0.001**
3–4	42 (35.3%)	66 (62.3%)	
N stage			
0–1	36 (30.3%)	21 (19.8%)	0.072
2–3	83 (69.7%)	85 (80.2%)	
Clinical stage			
II	22 (18.5%)	12 (11.3%)	** < 0.001**
III	82 (68.9%)	50 (47.2%)	
IVa	15 (12.6%)	44 (41.5%)	
Treatment			
CCRT	82 (68.9%)	72 (67.9%)	0.887
Non-CCRT	37 (31.1%)	34 (32.1%)	

*ECOG PS* Eastern clinical oncology group performance status, *BMI* body mass index, *CCRT* concurrent chemoradiation therapy.

Significant values are in bold.

### Prognostic value of FARI

**Figure 2:**
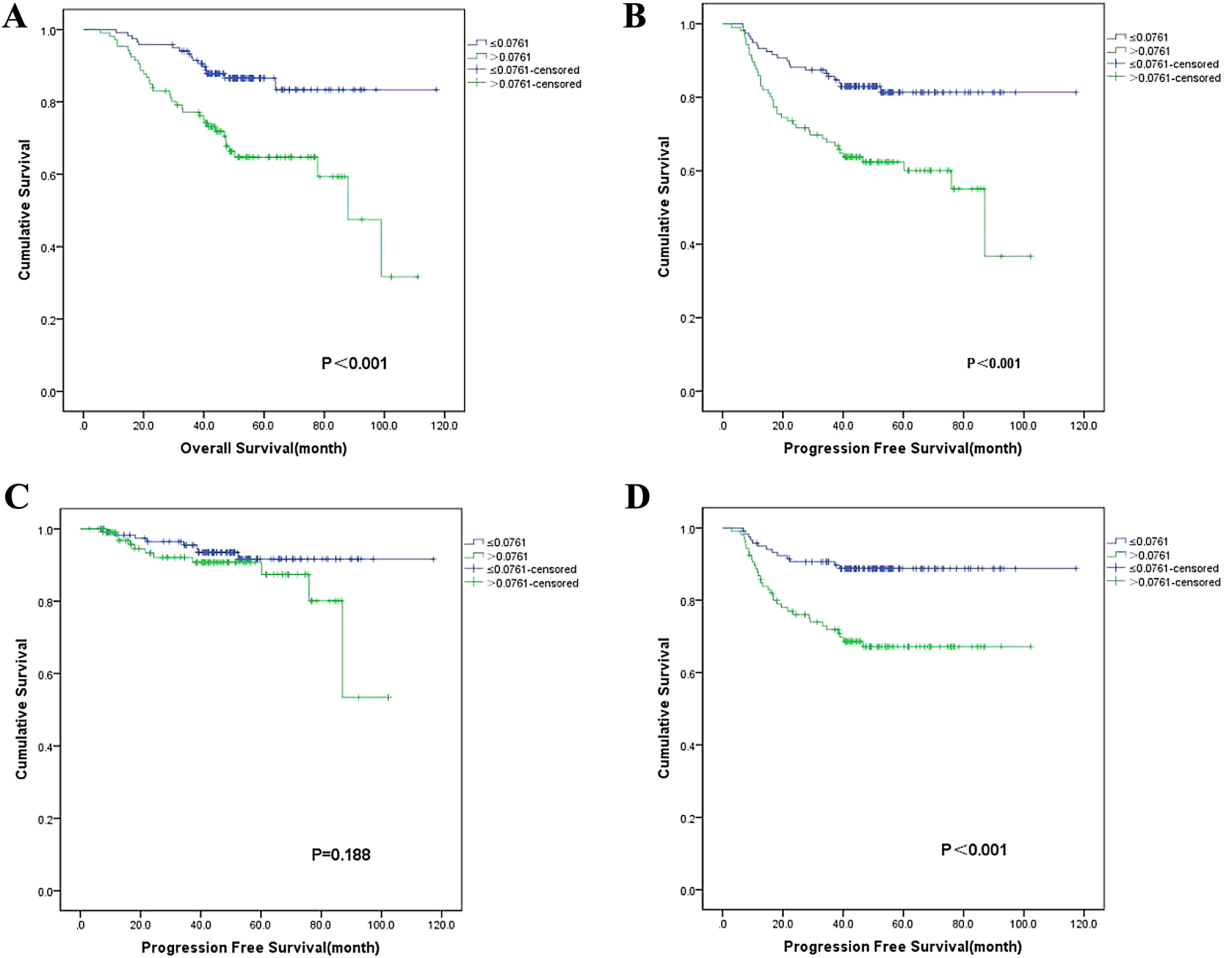
Kaplan–Meier survival curves of NPC patients (**A**–**D**). (**A**) Kaplan–Meier curves for OS according to FARI; (**B**) Kaplan–Meier curves for PFS according to FARI; (**C**) Kaplan–Meier curves for LRRFS according to FARI; (**D**) Kaplan–Meier curves for DMFS according to FARI; *OS* overall survival, *PFS* progression free survival, *LRRFS* local–regional relapse-free survival, *DMFS* distant metastasis-free survival.

Kaplan‒Meier analysis showed that FARI (p < 0.001), clinical stage (p = 0.010), T stage (p = 0.034), N stage (p = 0.022), and EBV-DNA (p = 0.025) were associated with patient OS. (Table 3, Fig. 2A). FARI (p < 0.001), clinical stage (p = 0.003), T stage (p = 0.012), N stage (p = 0.015), ECOG (p = 0.030), and EBV-DNA (p = 0.025) were associated with patient PFS (Table 3, Fig. 2B). Only clinical stage (p = 0.021).

**Table 3:** Univariate analysis of potential factors associated with OS, PFS, LRRFS, and DMFS.

Variables	OS			PFS			LRRFS			DMFS		
	Case	5y-(%)	P	Case	5y-(%)	P	Case	5y-(%)	P	Case	5y-(%)	P
Age												
≤ 60	196	77.3	0.227	196	72.9	0.550	196	91.2	0.882	196	78.7	0.827
> 60	29	69.0		29	69.0		29	91.7		29	78.4	
Sex												
Male	162	73.3	0.235	162	69.9	0.314	162	88.3	0.150	162	78.7	0.923
Female	63	83.3		63	78.8		63	98.1		63	78.8	
ECOG PS												
0	133	81.5	0.054	133	77.3	**0.030**	133	91.7	0.459	133	84.3	**0.015**
1–2	92	68.6		92	65.5		92	90.5		92	70.6	
BMI												
< 18.5	19	84.2	0.910	19	78.9	0.832	19	100.0	0.391	19	78.9	0.975
18.5–24.0	99	77.0		99	70.0		99	90.8		99	77.8	
> 24.0	107	73.9		107	71.5		107	90.2		107	79.3	
EBV-DNA (n = 142)												
< 400	81	82.7	**0.025**	81	80.2	**0.017**	81	95.9	0.095	81	86.1	**0.006**
≥ 400	61	65.9		61	60.4		61	87.3		61	64.4	
T stage												
1–2	117	82.7	**0.034**	117	79.3	**0.012**	117	94.1	0.059	117	85.1	**0.018**
3–4	108	69.4		108	65.0		108	88.0		108	71.4	
N stage												
0–1	57	85.4	**0.022**	57	83.2	**0.015**	57	98.1	**0.022**	57	84.8	0.120
2–3	168	73.1		168	68.7		168	88.5		168	76.6	
Clinical stage												
II	34	90.7	**0.010**	34	90.7	**0.003**	34	100.0	**0.021**	34	90.9	**0.012**
III	132	78.2		132	73.0		132	91.3		132	80.7	
IVa	59	63.7		59	60.8		59	85.1		59	66.9	
Treatment												
CCRT	71	84.6	0.067	70	78.7	0.153	71	90.9	0.975	71	86.8	**0.045**
Non-CCRT	154	72.1		155	70.1		154	91.5		154	74.8	
FARI												
High	106	64.7	** < 0.001**	106	62.4	** < 0.001**	106	90.8	0.188	106	67.1	** < 0.001**
Low	119	86.6		119	81.4		119	91.7		119	88.8	

*ECOG PS* Eastern clinical oncology group performance status, *BMI* body mass index, *CCRT* concurrent chemoradiation therapy, *OS* overall survival, *PFS* progression free survival, *LRRFS* local–regional relapse-free survival, *DMFS* distant metastasis-free survival.

Significant values are in bold.

and N stage (p = 0.022) were associated with patient LRRFS. (Table 3, Fig. 2C). FARI (p < 0.001), clinical stage (p = 0.012), T stage (p = 0.018), ECOG (p = 0.015), treatment modality (p = 0.045), and EBV-DNA (p = 0.006) were associated with patient DMFS (Table 3, Fig. 2D).

The variables that were found to be significantly correlated with patient prognosis, including clinical stage, ECOG, treatment modality, and FARI, were incorporated into the multivariate analysis. Cox regression analysis showed that clinical stage (HR 1.567, 95% CI 0.998–2.461, P = 0.051) and FARI (HR 2.399, 95% CI 1.294–4.450, P < 0.001) were independent prognostic factors of OS. Clinical stage (HR 1.607, 95% CI 1.050–2.461, P = 0.029) and FARI (HR 2.085, 95% CI 1.200–3.625, P = 0.009) were independent prognostic factors of PFS. Only clinical stage (HR 2.938, 95% CI 1.342–6.430, P = 0.005) was an independent prognostic factor of LRRFS. Clinical stage (HR 1.629, 95% CI 1.005–2.641, P = 0.048), treatment modality (HR 0.426, 95% CI 0.204–0.889, P = 0.023), and FARI (HR 2.527, 95% CI 1.288–4.958, P < 0.001) were independent prognostic factors of DMFS (Table 4).

**Table 4:** Multivariable Cox regression analyses for OS, PFS, LRRFS, and DMFS.

Variables	OS		PFS		LRRFS		DMFS	
	HR (95% CI)	P	HR (95% CI)	P	HR (95% CI)	P	HR (95% CI)	P
ECOG PS								
0			1.224 (0.724–2.071)	0.451			1.430 (0.773–2.647)	0.255
1–2								
Clinical stage								
II	1.567 (0.998–2.461)	**0.051**	1.607 (1.050–2.461)	**0.029**	2.938 (1.342–6.430)	**0.005**	1.629 (1.005–2.641)	**0.048**
III								
IVa								
Treatment								
CCRT							0.426 (0.204–0.889)	**0.023**
Non-CCRT								
FARI								
High	2.399 (1.294–4.450)	**0.005**	2.085 (1.200–3.625)	**0.009**			2.527 (1.288–4.958)	**0.007**
Low								

*ECOG PS* Eastern clinical oncology group performance status, *CCRT* concurrent chemoradiation therapy, *OS* overall survival, *PFS* progression-free survival, *LRRFS* local–regional relapse-free survival, *DMFS* distant metastasis-free survival.

Significant values are in bold.

## Discussion

In the present study, we evaluated the prognostic importance of FARI in a cohort of 225 NPC patients. The results showed that high FARI was related to unfavorable clinical characteristics and outcomes. Furthermore, FARI was an independent prognostic factor of OS, PFS, and DMFS. To the best of our knowledge, this is the first report on the prognostic role of FARI in NPC patients. This result indicated that FARI may be a promising blood-based prognostic index for NPC patients.

Increasing evidence has shown that the systemic inflammatory response^18–20^ and malnutrition^21–23^ play a critical role in the development and progression of various malignancies. Fibrinogen is a classical coagulation-related protein; however, an increasing number of studies have proven that it is also a marker of systemic inflammation and is involved in the progression of cancer via multiple mechanisms. Fibrinogen could impair macrophage migration and prevent fibrinogen-leukocyte interactions by mutating the leukocyte integrin binding motif on fibrinogen, which harms the antitumor immunity of the host^24^. Fibrinogen could also block the ability of NK cells to clear tumor cells, facilitating evasion of immune surveillance and metastasis^25^. Fibrinogen could promote epithelial-mesenchymal transition (EMT) via the AKT-mTOR pathway^8^. Preclinical research has proven that suppression of fibrinogen by miRNA could decrease the metastatic potential of tumor cells in mouse lung cancer models^26^. Thus, decreasing fibrinogen might be a potential strategy to improve outcomes in cancer patients by minimizing metastasis.

Malnutrition is a common complication of cancer that is related to poorer quality of life, more treatment interruption, and worse prognosis^27^. Cancer-related symptoms, such as dysphagia and bowel obstruction, as well as cancer-induced excess catabolism and inflammation, could significantly affect nutritional status^28^. Albumin (ALB) is a typical repetitiveness of nutritional status that is synthesized by the liver and suppressed by malnutrition and systemic inflammation^29^. In clinical studies,

ALB^30,31^ and ALB-related indices, including the CRP-albumin ratio^32^ and prognostic nutritional index^33^, have been proven to be independent prognostic factors in various cancers, including NPC.

FARI is a comparatively new marker of nutrition-inflammation status. Several studies have reported the prognostic value of FARI in many cancers, including esophageal squamous cell carcinoma^34^, hepatocellular carcinoma^35^, pancreatic neuroendocrine neoplasms^36^, and gastric cancer^37^. Although the cutoff value of FARI varies in different studies, all of the studies showed that elevated FARI is an adverse factor in cancer patients. This result indicated that fibrinogen and albumin have consistent effects in different cancer patients. Our group previously also reported that FARI is an independent prognostic factor of OS in lung adenocarcinoma patients^38^ and head and neck squamous cell carcinoma patients^13^. In the present study, we found that FARI is an independent prognostic factor of OS, PFS, and DMFS, but not LRRFS, in NPC patients. This result provides further support for the idea that the immune-inflammation status mainly affects the prognosis of cancer patients by influencing metastasis, not local relapse, which is in accordance with a previous report on mechanical and clinical research.

There are several limitations in this study. First, this is a single-center retrospective study with a long time span that may inherently carry some bias. Second, EBV-DNA has widely been accepted as a prognostic factor in Chinese patients. Some research has indicated that EBV-DNA > 4000 copies/ml predicts a worse prognosis in NPC patients^39^. However, the testing procedure of EBV-DNA has not been standardized thus far, which makes the results from different centers incomparable. In our center, the cutoff value of EBV-DNA is 400 ml/copy. In the present study, only 63.1% of the patients had an EBV-DNA test result. We found in univariate analysis that EBV-DNA was a prognostic factor with a cutoff value of 400 copies/ml. However, due to the limited sample size, it was not included in the multivariate analysis. Third, due to the limited sample size, no internal or external validation cohort was set to confirm the results. Therefore, more studies are still needed to further verify the clinical importance of FARI in NPC patients.

## Conclusions

In summary, a high FARI level is related to poorer prognosis, and FARI is an independent factor of OS, PFS and DMFS in NPC patients. It is an effective and economical marker that may facilitate prognosis stratification and indicate a new perspective in intervention strategies to improve the clinical outcomes of NPC patients.

## Data Availability

The raw data supporting the conclusions of this article will be made available by the corresponding author (email address: houtao@csu.edu.cn), without undue reservation.
